# Should we “eliminate” PDA shunt in preterm infants? A narrative review

**DOI:** 10.3389/fped.2024.1257694

**Published:** 2024-02-06

**Authors:** Aimann Surak, Amneet Sidhu, Joseph Y. Ting

**Affiliations:** ^1^ Division of Neonatal-Perinatal Medicine, Department of Pediatrics, University of Alberta, Edmonton, AB, Canada; ^2^ Division of Neonatal-Perinatal Medicine, Department of Pediatrics, McMaster University, Hamilton, ON, Canada

**Keywords:** PDA, preterm, ligation, piccolo, shunt

## Abstract

The patent ductus arteriosus frequently poses a significant morbidity in preterm infants, subjecting their immature pulmonary vascular bed to substantial volume overload. This, in turn, results in concurrent hypoperfusion to post-ductal organs, and subsequently alters cerebral blood flow. In addition, treatment has not demonstrated definitive improvements in patient outcomes. Currently, the optimal approach remains a subject of considerable debate with ongoing research controversy regarding the best approach. This article provides a comprehensive review of existing literature.

## Introduction

Patent ductus arteriosus (PDA) and its approach remain a topic of major controversy in the field of neonatology. This article reviews the best available literature around the topic with its limitations. We searched the PubMed database using controlled vocabulary and key words representing the concept “PDA” and “neonate”. Main articles were selected to be included by all authors.

## Why should we worry about patent ductus arteriosus in preterm infants?

**Figure 1:**
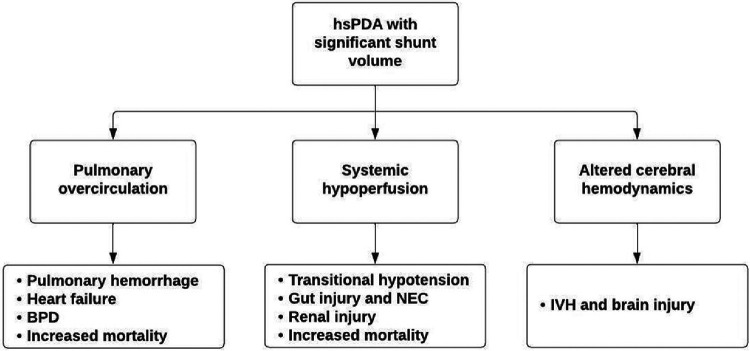
Pathophysiology of hsPDA.

The pathological entity of the PDA in preterm infants continues (1). PDA is linked to the most common preterm morbidities, including bronchopulmonary dysplasia (BPD) (2, 3), necrotizing enterocolitis (NEC) (4), etc. (Figure 1). Over the past decade, a shift of pendulum towards more conservative management, as opposed to pharmacological or surgical treatments has emerged (5). This trend is likely to be a response to potential side effects associated with pharmacological approach, as well as lack of marked inferiority in neonatal outcomes from conservative treatment (6–8) As an example, in the PDA-TOLERATE trial, preterm infants born <28 weeks’ gestation, were randomized to either early treatment, or to an observatory approach (9); there were no differences in primary outcomes (ligation or presence of a PDA at discharge), nor in secondary outcomes (NEC, BPD, BPD/death, weekly need for respiratory support) (9).

Early on in life, PDA plays a critical role during transitional circulation, and potentially contributes to dysregulated transitional hemodynamics such as intraventricular hemorrhage (10), and pulmonary hemorrhage (11).

PDA is associated with significant pulmonary morbidities and BPD. Animal studies (lambs with PDA) confirm considerable engorgement and increase in lymphatic conspicuity due to dilated architecture, leading to pulmonary edema and heart failure (12). Primate studies suggest that surgical closure of PDA may improve ventilation scores (13).

In humans, preterm infants born before 28 weeks’ gestation, when exposed to prolonged ductal shunt, this will contribute to the remodelling of pulmonary vasculature and subsequently, chronic pulmonary hypertension with BPD, and an increase in the BPD baseline rate in preterm infants exposed to PDA (2). In fact, this continues to be an issue in preterm infants who are discharged home with persistent PDA (14). Interestingly, it only takes 7–13 days of exposure to a moderate-to-large duct, for a significant increase in the incidence of BPD/death to become evident (15). PDA also plays a significant role in pulmonary hemorrhage pathophysiology; it appears that early treatment or prophylaxis, significantly reduce the incidence of pulmonary hemorrhage (11). In a recent Canadian study, infants who underwent PDA ligation, exhibited higher respiratory morbidities as early as the first few days of life (16). In this study, PDA ligation did not improve outcomes of death or BPD (16).

PDA also contributes to extra-pulmonary morbidities. There is a change in the shape and size of the myocardium, which peaks at 4 weeks of volume overload, potentially contributing to an increased risk of new-onset heart failure in adulthood (17). These findings correlate with an increased cumulative incidence of heart failure in preterm babies shown in a large Swedish population-based study (18), and the newly defined “preterm cardiomyopathy” (19–21). When hemodynamically significant, the PDA also affects the coronary arteries, by compromising coronary perfusion pressure and oxygen delivery to the myocardium in preterm infants (22).

The impact of PDA on preterm bowel, is evident, by impaired tissue oxygenation as observed in near-infrared spectroscopy (NIRS) studies (23), physiological post prandial superior mesenteric artery (SMA) flow (24), increase in mortality associated with NEC (4), and a five-fold increase in NEC (25). In addition, the incidence of renal injury increases with hemodynamic significant PDA (hsPDA), and renal saturation levels by NIRS less than 66% seem to be sensitive and specific indicators of hsPDA (26). Sellmer et al. showed that a large PDA, as early as day 3 of life, is associated with a two-fold increase in mortality, and a six-fold increase in the intraventricular haemorrhage (IVH) (25).

For long term outcomes associated with PDA, there is a lack of literature, and it is an area for future research. It appears that the long-term respiratory outcomes are related to BPD and its association with PDA (27). However, theoretically, the PDA would impact the developing brain in preterm infants, hence potentially contributing to worsening long term outcomes (28). There are few studies evaluated the long-term neurodevelopmental outcomes of PDA. In a multicenter cohort, Collins et al., did not find the PDA in premature infants to affect their neurodevelopmental outcomes at 3–18 years (29). Oncel et al., found no neurodevelopmental effects observed in preterm infants when evaluated with Bayley Scales of Infant Development II (Bayley-II), at the corrected age of 18–24 months (30). Similarly, Elbayiyev at al. found no association between hsPDA and poor neurodevelopmental outcomes, in a retrospective case control observational cohort (31). On the other hand, in a retrospective cohort of preterm infants born <29 weeks’ gestation, Janz-Robinson et al. suggested unfavorable neurodevelopmental course at 2–3 years of age, possibly related to PDA (32). Overall, this is an area which potentially needs to be further investigated.

## What is the definition of a hsPDA?

**Figure 2:**
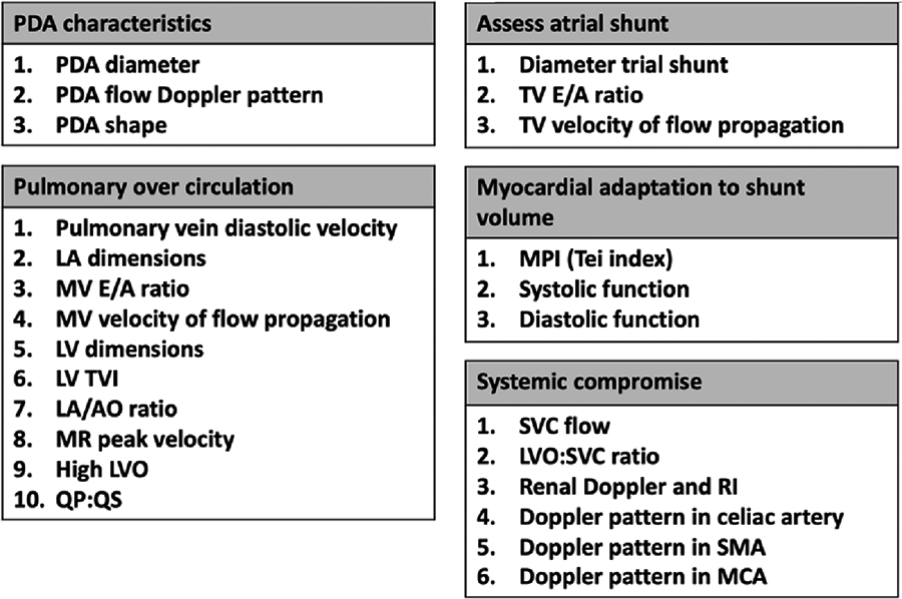
Targeted neonatal parameters needed to establish the hemodynamic significance of PDA.

Defining hsPDA is challenging due to the lack of a standardized consensus in literature (33, 34). Clinical assessment has been found to be neither sensitive nor specific, in predicting PDA shunt volume, particularly in the early days of life (35). Echocardiographic assessment scores have been developed (34, 36–38), most of them rely on similar parameters, such as size of PDA and evidence of left heart pressure and volume overloading (Figure 2).

Over the past two decades, the increasing application of targeted neonatal echocardiography (TnECHO), has provided a systematic approach to study the hemodynamic impact of PDA on circulation. This is through comprehensive assessment, which incorporates several domains (Figure 2), such as ductal size, flow Doppler pattern, and PDA shape (39). PDA is characterized by its length, width, tortuosity, and resistance to pharmacological closure (40). As a matter of fact, the PDA 3D structure is variable. While five types of ducts (labelled A-E) have been described, the increasing number of preterm infants referred for catheter closure, has led the identification of a additional type, known as the F type or fetal type ductus. This type is found mainly in prematurely born infants.

Evaluation of pulmonary circulation is achieved through analyzing multiple parameters obtained by TnECHO assessment (Figure 2). The preterm myocardium exhibits poor compliance due to its intrinsic characteristics, including a reduced number of calcium pumps, dependency on the L-type calcium channels, an underdeveloped sarcoplasmic reticulum and t-tubule system, disorganized mitochondria, and a higher proportion of non-contractile collagen and water in the myocardial interstitium (41). This makes it challenging for the myocardium to adapt to a high-volume ductal shunt.

Concurrently, the atrial shunt requires assessment, as it further enhance the pulmonary overcirculation, and subsequently, development of BPD (42–46). In addition, any evidence of a systemic compromise needs to be elaborated.

Recently, several cardiac biomarkers have been proposed for ductal assessment, particularly in resource-limited settings. B-type Natriuretic Peptide (BNP) and N-terminal-Pro-BNP (NTpBNP), traditionally used in adults to assess myocardial function and volume loading for prognostic identification post cardiac surgery, have gained increasing recognition in preterm infants. NTpBNP has shown promise as a potential screening tool for PDA, a marker for myocardial performance (47–51). Table 1 summarizes the most common suggested scoring tool used during TNE assessment (10). The application of a PDA score, defines hsPDA and guides management, and has demonstrated notable impacts on neonatal outcomes (10, 36). Also, such scores have been found to be reproducible (52).

**Table 1:** Suggested PDA scoring tool.

Measurement	0	1	2
Pulmonary vein d wave velocity (cm/s)	<30	30–50	>50
Mitral valve E wave velocity (cm/s)	<45	45–80	>80
LV IVRT (ms)	>50	30–50	<30
LA:AO ratio	<1.3	1.3–2.2	>2.2
LVO:RVO	<1.5	1.5–2.0	>2
Aortic/Peripheral Doppler flow reversal	Forward/Absent		Reversed
Ductus diameter indexed to weight (mm/kg)	<1.5	1.5–3.0	>3

## Why is there a lack of correlation between PDA treatment and improved neonatal outcomes?

Existing literature about PDA management in preterm infants, did not discernibly show improved neonatal outcomes. One example is the recent BeNeDuctus Trial (53, 54), showing that expectant management of PDA in preterm infants, was not inferior to early ibuprofen treatment with respect to neonatal outcomes (53, 54). In this trial, a total of 273 infants were randomized to receive either expectant management or early treatment with ibuprofen. Authors found that the expectant management is not inferior to the treatment when assessing the composite primary outcome of necrotizing enterocolitis, moderate to severe BPD, or death at 36 weeks’ postmenstrual age [46.3% vs. 63.5%, absolute risk difference, −17.2 percentage points; upper boundary of the one-sided 95% confidence interval (CI), −7.4; *P* < 0.001 for noninferiority] (53, 54).

Such lack of correlation in literature, between treating PDA and improved outcomes, is often subject to criticism, and it could be attributed to various factors (Table 2):

1- Absence of a standardized methodology for defining hsPDA across the literature. Roughly, 40% of the trials omitted any echocardiography data assessment (55). In most instances, hsPDA was primarily defined based on its dimeter alone, which has weak correlation with echocardiographic markers of shunt volume (56). Another example is Early PARacetamol Trial (EPAR) (57), preterm infants born at <29 weeks’ gestation, were randomized to receive early treatment with acetaminophen or placebo, based on ductal diameter >0.9 mm at 6 h of life (57). In addition, it is noteworthy that PDA diameter has significant inter-observer variability in 2D and color Doppler in preterm infants (58), and the PDA image on 2D view, does not accurately represent the PDA as a 3D structure, and it could potentially over- or underestimate ductal diameter (40). This highlights the importance of comprehensive echocardiographic evaluation, to provide a better understanding of the hemodynamic consequences of PDA.
2- Notable heterogeneity in the inclusion criteria, as well as the analyzed of outcomes among created difficulties for direct comparison. Variable outcomes were analyzed, and BPD is often an outcome in PDA-related literature (59, 60); few studies analyzed neurodevelopmental outcomes (29–32, 61), and others assessed composite outcomes of NEC, BPD, or death (53, 54). A standardized contemporary framework in PDA care that \supports the practice of evidence-based medicine is necessary (62).
3- Ductal shunt was not completely eliminated in the intervention arm in most studies, which leads to ongoing exposure to ductal shunt. Generally, the rate of ductal closure remains around 60%–70%, attributable to the partial effectiveness of pharmacotherapy as compared to surgical closure (63–65).
4- Lack of equipoise: In the control arm of many studies, almost two-thirds of infants received a rescue treatment. For example, in the DETECT trial, preterm infants born <29 weeks’ gestation were screened for a large PDA and randomized to receive either indomethacin or placebo before age 12 h of life (11). In the placebo arm, 40% of infants received an open-label treatment (11). This emphasizes the necessity of upholding equipoise in well-designed randomized controlled trials, a sentiment echoed by the Committee on Fetus and Newborn by American Academy of Pediatrics (66).

**Table 2:** Issues and deficits in available literature.

1. No standardized consensus defining the hemodynamic significance
2. Evidence is not contemporary
3. Shunt was not completely eliminated
4. Lack of equipoise

## Ductal shunt limitation vs. elimination

Current practice when managing hsPDA, includes several pharmacological, non-pharmacological, and surgical interventions (67). The approach of limiting the ductal shunt, often referred to as conservative management, focuses on modulating the factors that dictate the shunt volume. Typically, pharmacological interventions, employing non-steroidal anti-inflammatory drugs (NSAIDs) or acetaminophen, induce ductal constriction (38, 68–71). Nonetheless, this does not assure complete ductal closure or shunt elimination, even when combined, with a success rate hovering around 60%–70% in most scenarios (38, 69–72). It is also worth mentioning the recent systematic review and meta-analysis regarding the high-dose ibuprofen, which seems to be more effecting compared to standard-dose ibuprofen, but still did not significantly decrease the failure rate of PDA closure in preterm infants after the first course (Relative risk (RR) 0.74, 95% confidence interval (CI) 0.53 −1.03, 6 studies, *N* = 369) (73).

A common clinical practice is regulating the systemic-pulmonary pressure gradient by increasing the pulmonary vascular resistance (by maintaining mean airway pressure) could be considered by optimizing the mean airway pressure and allowing for permissive hypercapnia (5, 74). When it comes to fluid restriction, clinicians should exercise caution with this practice (75–77), given most of trials are non-contemporary, and conducted in moderately preterm infants, which may not be applicable to extremely preterm infants currently (67).

Another strategy entails enhancing hemoglobin by packed red blood cell transfusion to limit the ductal shunt (78). In theory, blood transfusions can elevate blood viscosity, which may help in reduction of ductal shunt volume (79).

For definitive ductal shunt elimination, the only two strategies are surgical ligation, and percutaneous catheter closure. While PDA ligation ensures immediate shunt elimination, it is associated with unfavorable morbidities, and potentially long-term side effects. This encompasses post-ligation cardiac syndrome and respiratory failure, an increased risk of BPD with early ligation, vocal cord paresis retinopathy of prematurity, and neurodevelopmental impairment (80–84).

Recently, Food and Drug Administration (FDA) approved the use of the “The Amplatzer Piccolo” device for PDA closure in preterm infants. This was based of its proven efficacy and safety in this vulnerable population (85). This approach seems to be gaining popularity, as it is feasible, effective, and relatively safe (86, 87). It provides a definitive and complete ductal shunt elimination with improvement of respiratory status following the procedure (88). In a recent meta-analysis by Bischoff et al., this approach was feasible in infants ≤1.5 kg with only few major adverse events with high rate of success (89).

In fact, it can be utilized in preterm infants as small as 700 g, and as early as 3–4 weeks (85, 86). The left pulmonary stenosis and migration of the device are potential complications to this procedure (85, 86). Anecdotal data showed that the incidence of cardiorespiratory instability, might be less common with device closure as compared to ligation (40, 90, 91). The comparatively favorable side effects profile of device-closure versus ligation likely explains the decline in the rates of surgical ligation (86).

## Future directions and ideal study design

There is no controversial topic in the neonatal field like the PDA approach and its management. This continues to be a the most contentious topics in the care of preterm infants (92). Currently, there is no consensus about the ideal treatment. Catheter closure ensures a complete ductal shunt elimination (as opposed to limiting or reducing it), aligning more closely with the ideal goal of treatment; however, more research is needed to delineate safety profile. Future trials should consider randomizing infants with hsPDA to a complete shunt elimination vs. other approaches. Percutaneous Intervention Versus Observational Trial of Arterial Ductus in Low weight Infants (PIVOTAL) is an ongoing trial, where a complete shunt elimination would be compared to observational approach (https://www.pivotalstudy.org). in addition, emphasis should be put on standardized definitions of hsPDA with validation of the echocardiographic markers. Precise definition of outcomes in these trials is equally important.

## Limitation of this review

This article is a general overview of the available literature pertaining the topic of PDA in preterm infants. There is a significant degree heterogeneity in the literature making a structured methodological search difficult. Since, the review is written by 3 authors who received similar structured training in TnECHO, and currently practicing in similar tertiary care neonatal settings in Canada, there might be an element of potential bias.
